# Suppressed activation of the *IRF7* and *TLR9* by *JAK2*V617F gold nanoparticles

**DOI:** 10.1007/s00251-025-01374-y

**Published:** 2025-02-28

**Authors:** Berkay Tokcan, Esra Nur Demirtaş, Selçuk Sözer

**Affiliations:** 1 Department of Genetics, Aziz Sancar Institute of Experimental Medicine, Istanbul University, Gureba Str., Capa Campus, No:69, 34093 Sehremini/Istanbul, Türkiye; 2 Institute of Health Sciences, Istanbul University, 34093 Istanbul, Türkiye

**Keywords:** *TLR9*, *IRF7*, IL-8, CGAS-STING, Myeloproliferative neoplasms, *JAK2*V617F, Gold nanoparticles

## Abstract

Philadelphia chromosome-negative myeloproliferative neoplasms (Ph-MPNs) are characterized by the overproduction of myeloid cells and a lack of response to cytokine signaling, along with genomic instability and the accumulation of nucleic acids in the cytoplasm. In this study, we investigated the effects of oligonucleotide-gold nanoparticle conjugates (ON-GNPs) targeting *JAK2* or *JAK2*V617F mRNAs on nucleic acid-sensing pathways in HEL, SET2, and K562 cell lines. We evaluated changes in gene expression related to TLR9 and cGAS/STING pathways, RAGE/TLR9 receptor dynamics, and inflammatory cytokine release over short-term (0.5–2 h) and long-term (24–72 h) exposures. Our results demonstrated that ON-GNPs transiently suppressed *TLR9*, *IRF7*, and *NFKB1* expression during the short term, followed by significant upregulation after 24 h, persisting up to 72 h. Notably, *JAK2*V617F-targeting ON-GNPs induced heightened *IRF7* activation in HEL and SET2 cells after 24 h without affecting TLR9/RAGE expression. Additionally, IL-8 secretion increased in HEL and SET2 culture media after 72 h, correlating with interferon pathway activation. This study reveals that complementary ON-GNPs can modulate nucleic acid-sensing pathways, suppressing IL-8 and inflammatory signaling in the short term while inducing delayed activation of *TLR9* and *IRF7* in the presence of *JAK2*V617F. These findings provide a promising foundation for developing ON-GNP-based therapeutic strategies to manage inflammation and disease progression in Ph-MPNs.

**Supplementary Information:**

The online version contains supplementary material available at 10.1007/s00251-025-01374-y.

## Introduction

Philadelphia chromosome-negative myeloproliferative neoplasms (Ph-MPNs) are clonal hematopoietic stem cell disorders characterized by the excessive production of blood cells belonging to the myeloid lineage (Nangalia and Green 2017; Tefferi and Pardanani 2015). The *JAK2*V617F mutation, a single-nucleotide substitution (c.1849G > T) in the *JAK2* gene, results in a constitutively active JAK2 variant (Kralovics et al. 2005). This leads to increased cell proliferation and survival while diminishing sensitivity to cytokines (Wang and Zuo 2019). Beyond its direct effects on hematopoiesis, recent studies highlight the broader impact of *JAK2*V617F on immune cell function and cytokine production, revealing its complex role in modulating inflammatory responses (Kleppe et al. 2018; Dunbar et al. 2023). Chronic inflammation not only interacts with *JAK2*V617F-driven pathways but also plays a pivotal role in disease progression and clonal evolution in MPNs (Gou et al. 2022; Hermouet 2023; Lussana and Rambaldi 2017). The constitutive activation of JAK2 in *JAK2*V617F-positive cells disrupts normal cellular processes, contributing to genomic instability. This instability promotes the accumulation of cytosolic nucleic acid fragments, which act as ligands for nucleic acid-sensing pathways. The activation of these pathways drives sustained inflammatory responses, exacerbating disease symptoms and highlighting their central role in MPN pathophysiology.

Toll-like receptors (TLRs), particularly TLR9, recognize pathogenic and endogenous nucleic acids and play key roles in both innate and adaptive immunity (Baris et al. 2021; Schlee and Hartmann 2016). TLR9, found on the intracellular membrane, detects DNA with CpG motifs and RNA–DNA hybrids, facilitating the production of pro-inflammatory cytokines and type I interferon (Schlee and Hartmann 2016; Kumagai et al. 2008). The receptor for advanced glycation end products (RAGE) promotes nucleic acid uptake through the endosomes where TLR9 is located, enhancing TLR9-dependent responses and activating the *NF-κB* transcription factors crucial for immune modulation in cancer and chronic inflammation (Sirois et al. 2013; El-Zayat et al. 2019; Yan et al. 1994; Chuang and Ulevitch 2000; Karapetyan et al. 2020; Chen et al. 2013). Additionally, the cyclic GMP-AMP synthase (cGAS)-stimulator of interferon genes (STING) pathway is vital for detecting cytoplasmic DNA, including damaged or foreign genetic material, and initiating the immune response (Hopfner and Hornung 2020; Ma et al. 2021; Ishikawa and Barber 2008) leading to the expression of type I interferons, pro-inflammatory cytokines, and non-canonical NF-κB signaling (Yum et al. 2021; Tanaka and Chen 2012). These processes are essential for promoting hematopoietic cell proliferation and differentiation and preventing tumor formation in hematopoietic stem cells (Liao et al. 2020).

Although a correlation exists between malignancy and nucleic acid-sensing pathways, the involvement of RAGE, TLR9, and cGAS-STING in Ph-MPNs is limited. Considering the consequences of dysregulated inflammation and genome instability in MPNs, our study aimed to examine the potential involvement of nucleic acid-sensing pathways, particularly TLR9 and cGAS-STING pathways, in a *JAK2*V617F-positive environment and propose a novel treatment modality for MPNs.

Oligonucleotides (ONs) conjugated to spherical gold nanoparticles (GNPs) enhance stability and cellular uptake through endocytic mechanisms without affecting cell viability (Choi et al. 2013; Mirkin and Petrosko 2023; Cutler et al. 2012; Kapadia et al. 2018; Sozer et al. 2019; Rosi et al. 2006). These properties make ON-GNPs a promising platform for therapeutic applications, particularly in targeting nucleic acid-sensing pathways and modulating gene expression. In this study, we investigated the potential of ON-GNPs to interfere with key inflammatory regulators, including the TLR9 and cGAS/STING pathways, which play critical roles in innate immune responses and inflammation. Additionally, we designed ONs to specifically hybridize with the mRNA sequences of *JAK2* and *JAK2*V617F, aiming to reduce the aberrant mRNA levels associated with Ph^−^MPN pathology. This hybridization not only inhibits the translation of these mRNAs into JAK2 proteins but may also influence other, as-yet-unidentified cellular processes mediated by these hybridized transcripts.

In this study, we utilized the HEL and SET2 cell lines, which are known to carry biallelic or monoallelic *JAK2*V617F, respectively, as models for Ph-MPN. By investigating SET2 and HEL cell lines, which resemble essential thrombocythemia (ET) and polycythemia vera (PV), respectively, we gained insights into the differential inflammatory responses associated with these distinct MPN subtypes. Additionally, the chronic myeloid leukemia cell line K562, which expresses BCR/Abl fusion protein but lacks *JAK2*V617F, was included for comparative analysis. The cells were treated with ON-GNPs carrying complementary sequences targeting either *JAK2* or *JAK2*V617F mRNA. Treatments were conducted over both short-term (0.5–2 h) and long-term (24–72 h) durations. Following these treatments, we analyzed the expression levels of key genes involved in inflammatory and nucleic acid-sensing pathways, *TLR9*, *IRF7*, *NFKB1*, *cGAS*, *STING*, *JAK2*, *TBK1*, *IRF3*, and the release of pro-inflammatory cytokines under the experimental conditions.

The administration of ON-GNPs into cells demonstrated a dual-phase effect on the expression of key genes involved in inflammatory signaling pathways, including *IRF7*, *TLR9*, and components of the cGAS/STING pathway. During short-term treatments, these pathways were significantly suppressed, with the inhibitory effect persisting up to 24 h. However, under specific conditions in cell lines harboring the *JAK2*V617F mutation, a delayed upregulation of *IRF7* and *TLR9* expression, accompanied by increased IL-8 secretion, was observed at later time points. This biphasic response was independent of the RAGE/TLR9 pathway, suggesting the involvement of alternative regulatory mechanisms in modulating nucleic acid-sensing pathways. The initial suppression induced by ON-GNPs may recalibrate dysregulated cytokine production, mitigate genomic instability, and influence the hematopoietic stem cell niche in MPNs. The subsequent upregulation of inflammatory mediators highlights the complex interplay between *JAK2*V617F-driven inflammation and nucleic acid sensing, underscoring the potential of ON-GNPs as a precision therapeutic tool for managing the chronic inflammation and disease progression associated with MPNs.

## Material and method

### Incubation of cell lines with oligonucleotide carrying gold nanoparticles

Three different cell lines were used in the experiments: the human erythroleukemia cell line (HEL), which has biallelic *JAK2*V617F and is maintained in RPMI-1640 supplemented with 10% fetal bovine serum (FBS) and 1% penicillin/streptomycin (P/S) (100 U/mL, 100 µg/mL). The human megakaryoblastic cell line (SET-2) carrying monoallelic *JAK2*V617F was maintained in RPMI 1640 containing 20% FBS and 1% P/S. The human chronic myelogenous leukemia cell line (K562) did not carry *JAK2*V617F and was maintained in IMDM containing 10% FBS and 1% P/S. HEL and SET2 were obtained from DSMZ (Leibniz Institute DSMZ, Leibniz, Germany), and Istanbul University AS-DETAE, Cell Culture Laboratory, provided the K562 cells. All the cell lines were incubated at 37 °C in 5% CO2 at ≥ 95% humidity.

The AuNPs were custom-designed with a diameter of ~ 13 nm (Yue et al. 2018) and functionalized with oligonucleotide (ON) chains, with an average length of 80 ± 10 bases. The resulting ON-GNPs had a final diameter of ~ 25 nm, with a surface density of around 1.7–2.0 × 10^13^ chains/cm^2^ (Giljohann et al. 2007).

ON-GNP complementary transcripts were tested against either *JAK2* or *JAK2V617F* (Exicure, Chicago, IL, USA). The ON sequences were listed in Supplement Table 1. A scramble control, which does not complement any transcript in situ, was included in the experiments. ON-GNPs were applied to cells according to the manufacturer’s protocol. Briefly, cells were transferred to a 24-well culture plate at 300,000 cells/well in 500 µL of growth medium with a 200 pM probe in a humidified environment at 37 °C with 5% CO2 for incubation. Cells were collected in 0.5, 1, 1.5, 2, 24, 48, and 72 h.

### mRNA isolation and real-time quantitative RT-PCR

The collected cells were used to obtain the total mRNA for each condition. A total RNA Purification Kit (Jena Bioscience, Jena, Germany) was used for RNA isolation, according to the manufacturer’s protocol. The purity and concentration of the RNA extracts were measured using a NanoDrop 2000 spectrophotometer (Thermo Fisher Scientific, USA). The collected RNA was converted into first-strand complementary DNA (cDNA) and synthesized from 100 ng of total RNA using a SCRIPT cDNA Synthesis Kit (Jena Bioscience, Jena, Germany) and a qPCR SybrMaster kit (Jena Bioscience, Jena, Germany) according to the manufacturer’s protocol.

The primers for the transcripts that were studied, including *TLR9*, *IRF7*, *NFKB1*, *cGAS*, *STING*, *JAK2*, *TBK1*, *and IRF3*, are provided in Supplement Table 1. A real-time quantitative RT-PCR instrument LightCycler II 480 (Roche, Switzerland) was used for 45 cycles, with each cycle having 10 s of denaturation at 95 °C, 10 s of hybridization at 60 °C, and 10 s of elongation at 72 °C as the reference gene *ACTB* was applied.

### Flow cytometric analysis of cells

After incubation at 37 °C in 5% CO_2_ with ≥ 95% humidity with ON-GNPs, cell lines were collected on specified time points, and following centrifugation, the cells were resuspended in 100 µL of buffer containing phosphate-buffered saline (PBS) with 7.5% bovine serum albumin + 0.5 M EDTA and labeled with anti-RAGE antibody for the cell surface analysis with 0.1% PI in buffer. Intracytoplasmic detection of both RAGE and TLR9 was performed using the IntraPrep Permeabilization Reagent (A07803, Beckman Coulter Life Sciences, Indianapolis, IN, USA), as suggested by the manufacturer, with mAbs for RAGE (FAB11795R, anti-Human RAGE-AlexiaFlour-647; R&D Systems, Minneapolis, MN, USA) and TLR9 (S16013D, PE-anti-Human/CD289, BioLegend, San Diego, CA, USA). The cells were washed twice with buffer and analyzed using a FACSCalibur instrument (Becton Dickinson Biosciences, Franklin Lakes, NJ, USA).

### Measurement of culture medium cytokine concentrations

Cytokine concentrations in the culture medium of HEL and SET2 cells after 24, 48, and 72 h of incubation with ON-GNPs were measured using LEGENDplex™ Human Inflammation Panel-1 with V-bottom Plate (BioLegend, San Diego, CA, USA) according to the manufacturer’s protocol. The cytokine panel included IL-1β, IFN-α2, IFN-γ, TNF-α, MCP-1 (CCL2), IL-6, IL-8 (CXCL8), IL-10, IL-12p70, IL-17A, IL-18, IL-23, and IL-33. After each incubation period with ON-GNPs, the supernatant of the growth medium of the cells was collected, and a cytokine assay was performed separately. Data acquisition and analysis were performed using a FACSCalibur instrument.

### Statistical analysis

Two replicate wells and two separate RT-PCR runs were performed for each condition. The 2^−(ΔΔCt)^ method calculated average relative gene expression by ΔCt values of untreated controls were extracted from treated conditions and converted the values to relative fold changes. GraphPad Prism v.8 (GraphPad Prism Inc., San Diego, CA, USA) was used for statistical analysis and plotting of expression graphs. Two-tailed ANOVA was used to determine the statistical significance between groups with a threshold *p*-value, which is presented in each figure legend and the text.

## Results

### Delayed activation of inflammatory pathway-related genes with complementary JAK2 and JAK2V617F attached gold nanoparticles

**Fig. 1:**
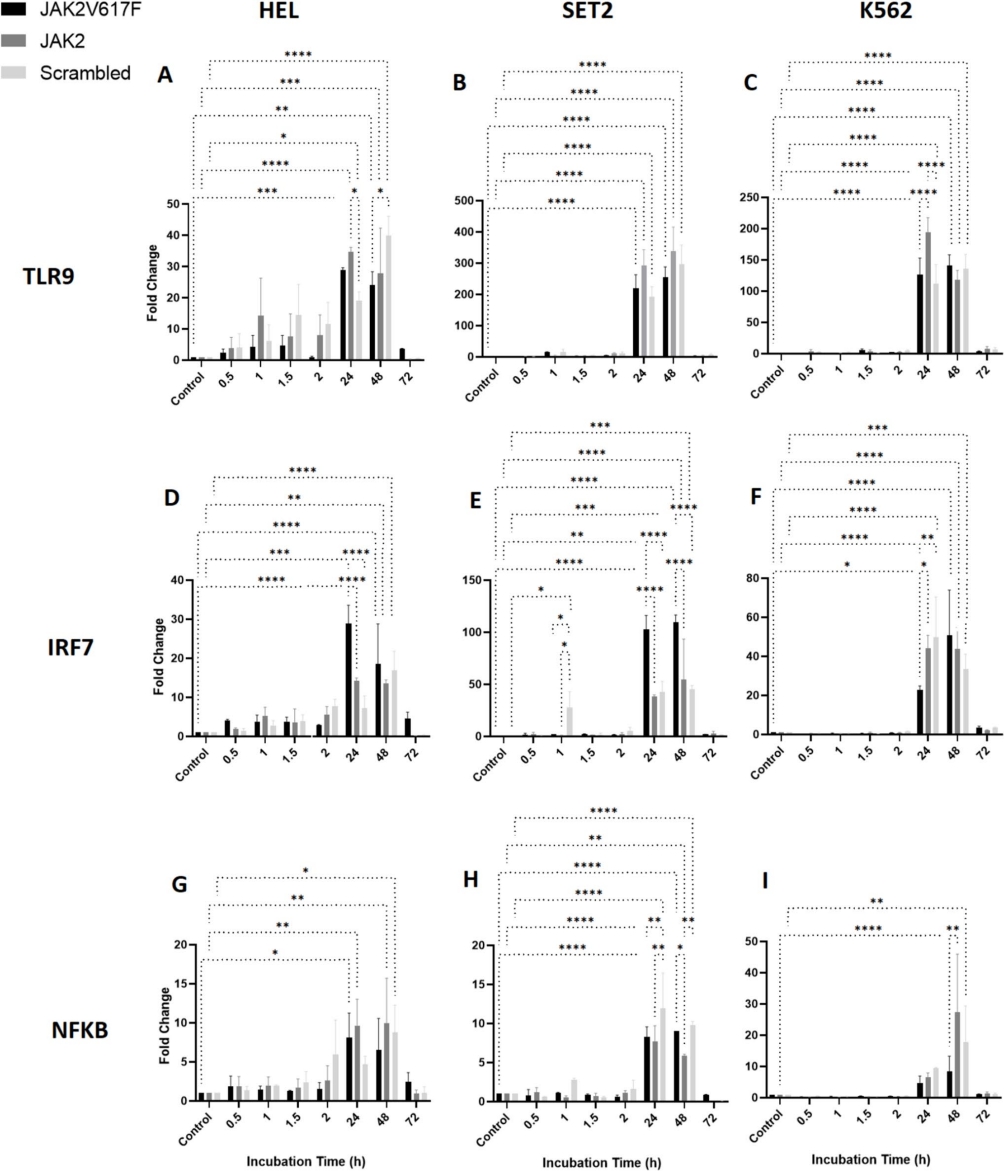
Relative expressional change of *TLR9*, *IRF7*, and *NFKB1* genes upon varying types of GNP application (complementary to JAK2V617F mRNA-specific, JAK2 mRNA-specific, and scrambled-non-specific to any mRNA) in cell lines of *JAK2*V617F biallelic HEL (**A**, **D**, **G**), *JAK2*V617F monoallelic SET2 (**B**, **E**, **H**), and *JAK2*V617F-negative K562 (**C**, **F**, **I**) for short-term (0.5–2 h) and long-term (24–72 h) durations (*p < 0.05, **p < 0.01, ***p < 0.001, ****p < 0.0001)

Investigation of the *TLR9* expression across HEL, SET2, and K562 cell lines revealed dynamic response when treated with ON-GNPs targeting wild-type *JAK2* (*JAK2*), *JAK2*V617F, and scramble control. The relative expression of *TLR9* was assessed at short-term (0.5–2 h) and long-term (24–72 h) time points, as shown in Fig. 1A, B, and C. Overall, *TLR9* expression remained stable across all treatments during the first 24 h. However, a significant increase in *TLR9* expression was observed between 24 and 48 h, which then returned to baseline levels by 72 h of incubation.

The most pronounced change in *TLR9* expression was detected in SET2 cells, where a substantial increase (~ 200–300-fold, *p* < 0.0001) occurred at 24 and 48 h before sharply declining at 72 h (Fig. 1B).

This suggests a cell type-specific response mechanism in which the megakaryoblastic lineage of SET2 cells may enhance their sensitivity to ON-GNP treatment in the long term. Differences in transcriptional regulation, nucleic acid-sensing pathway activity, and cytokine feedback loops may contribute to this heightened response. In contrast, HEL cells (erythroblastic) and K562 cells may rely on distinct regulatory mechanisms that modulate inflammatory signaling differently. Interestingly, no significant differences in relative *TLR9* expression were observed between treatments with ON-GNPs targeting *JAK2*, *JAK2*V617F, or the scramble control, indicating that the observed effects are likely not dependent on the specific target of the ON-GNPs.

A parallel assessment of relative *IRF7* expression under similar experimental conditions (Fig. 1D, E, and F) revealed minimal changes during the early phase (0.5–2 h) of exposure. However, a significant increase in *IRF7* expression was detected at 24 and 48 h and subsequently normalized by 72 h. Notably, *JAK2*V617F-GNPs induced a markedly higher expression of *IRF7* in HEL and SET2 cells during these intervals with fold changes of approximately 20 (*p* < 0.001) and 100 (*p* < 0.0001), respectively. This pronounced response highlights the potential influence of genetic variations, such as the presence of *JAK2*V617F, on cellular responses to ON-GNP treatment (Fig. 1D, E).

The marked upregulation of *IRF7*, particularly in *JAK2*V617F-positive HEL and SET2 cells, highlights the critical role of type I interferon responses in nucleic acid sensing within the context of MPNs. This finding aligns with previous reports of heightened interferon signaling associated with *JAK2*V617F-driven inflammation. Overall, the transient activation of *TLR9* and *IRF7*, followed by their normalization, suggests that ON-GNPs may recalibrate dysregulated inflammatory pathways in MPNs, reducing chronic inflammation without prolonged immune suppression.

Investigations on *NFKB1* expression revealed interesting results. In all cell lines, short-term exposure (0.5–2 h) to ON-GNPs had no effect; however, a notable increase was observed at 24 h across all ON-GNP treatments, which diminished by 72 h. HEL and SET2 cells exhibited an earlier but transient increase in *NFKB1* expression significantly elevated at both 24 h and 48 h (~ tenfold) in response to *JAK2*- and *JAK2*V617F-GNPs before declining (Fig. 1G, H). Interestingly, K562 cells displayed a delayed and selective response, with *NFKB1* upregulation occurring only at 48 h, showing a ~ 30-fold increase (*p* < 0.001) with *JAK2*-GNP and a ~ 20-fold increase (*p* < 0.001) with scramble-GNP (Fig. 1I). The significant increase in *NFKB1* expression following *JAK2*-GNP treatment suggests a potential interplay between *JAK2*-targeting ON-GNPs and BCR-ABL-driven pathways. This effect may arise from off-target interactions, secondary inflammatory signaling activation, or indirect modulation of NF-κB via altered cytokine dynamics.

### Delayed activation of cGAS-STING pathway with complementary JAK2 and JAK2V617F mRNAs attached gold nanoparticles

To investigate the role of alternative nucleic acid-sensing pathways in intracytoplasmic activities, we analyzed the expression of key cGAS/STING pathway genes, including *cGAS*, *STING*, *TBK1*, and *IRF3*, in response to ON-GNP treatment.

**Fig. 2:**
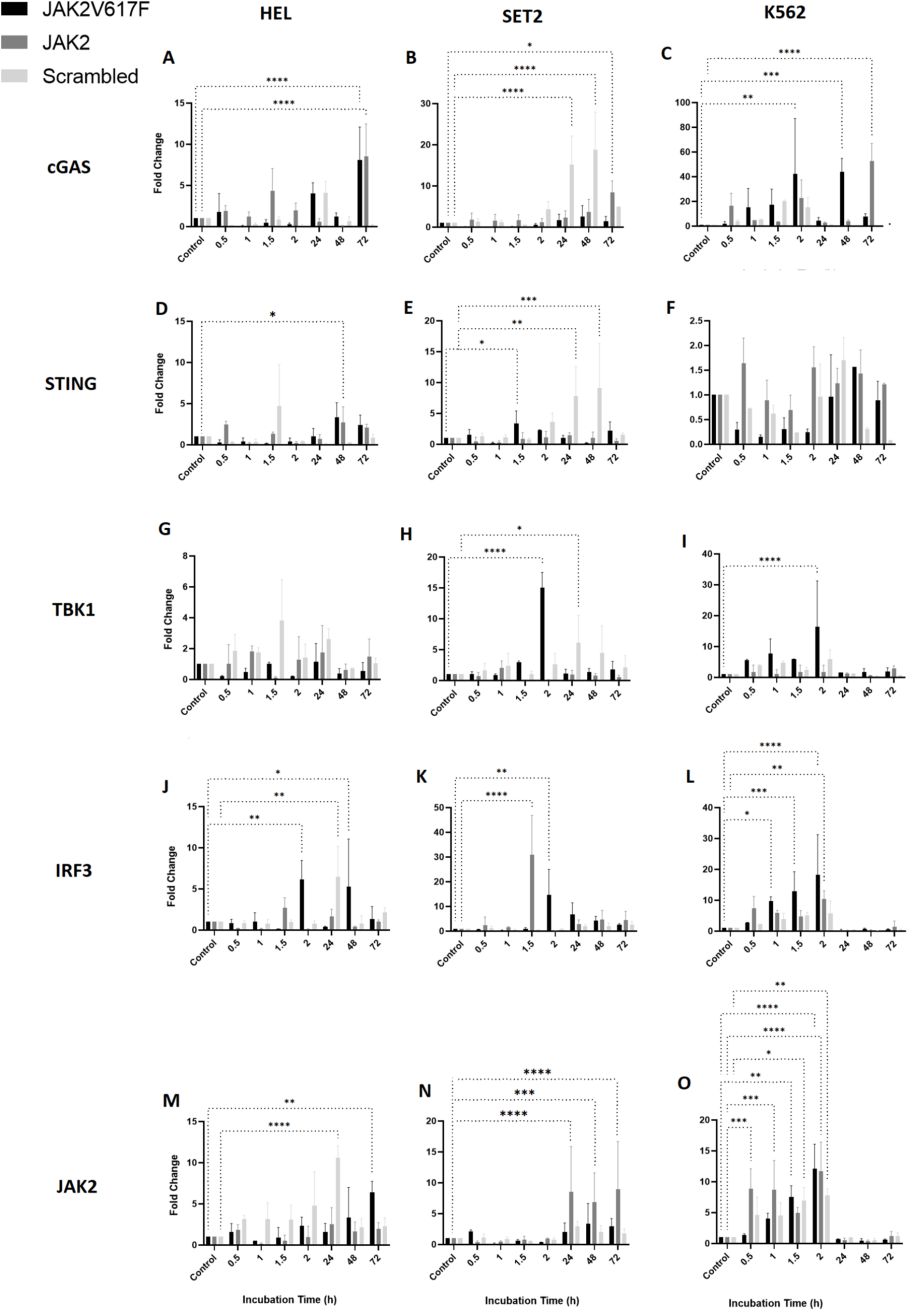
Relative expressional change of *cGAS* (**A**, **B**, **C**), *STING* (**D**, **E**, **F**), *TBK1* (**G**, **H**, **I**), *IRF3* (**J**, **K**, **L**), and *JAK2* (**M**, **N**, **O**) in cell lines HEL, SET2, and K562 upon incubation with *JAK2*, JAK2V617F, and scramble-GNPs for short-term (0.5–2 h) and long-term (24–72 h) durations. (*p < 0.05, **p < 0.01, ***p < 0.001, ****p < 0.0001)

Prolonged exposure (72 h) to ON-GNPs significantly increased cGAS expression across all three cell lines as shown in Fig. 2A–C. HEL cells showed a marked upregulation of cGAS with both *JAK2*-GNP (*p* < 0.001) and *JAK2*V617F-GNP (*p* < 0.0001) (Fig. 2A). In SET2 cells, a significant increase was observed only with scramble-GNP at 24 h and 48 h (*p* < 0.0001) and *JAK2*-GNP at 72 h, while *JAK2*V617F-GNP elicited no substantial effect (Fig. 2B). In K562 cells, relative *cGAS* expression profoundly increased (~ 40-fold, *p* < 0.01) at 2 h and 48 h with *JAK2*V617F-GNP (*p* < 0.001) and at 72 h with *JAK2*-GNP (*p* < 0.0001) (Fig. 2C). These findings suggest a cell line- and treatment-specific regulation of cGAS expression. However, *STING* expression remained mostly unaffected by ON-GNP treatment, with isolated exceptions. In HEL cells, STING was significantly upregulated at 48 h with *JAK2*V617F-GNP (*p* < 0.05) (Fig. 2D), and in SET2 cells, *STING* expression increased at 1.5 h with the *JAK2*V617F-GNP (*p* < 0.05) and at 24 h (*p* < 0.01) and 48 h (*p* < 0.001) with scramble-GNP (Fig. 2E). In K562 cells, no substantial *STING* response was detected despite strong cGAS induction (Fig. 2F). These results indicate that in SET2 cells, *STING* activation occurs only in response to non-specific ON-GNP treatment (scramble-GNP), whereas *JAK2-* and *JAK2*V617F-targeting ON-GNPs had no effect on *STING* expression.

*TBK1* expression was generally stable, with minor exceptions, such as increased expression in SET2 at 2 h with *JAK2*V617F-GNP (*p* < 0.001) and in K562 at 2 h with *JAK2*V617F-GNP (*p* < 0.001) (Fig. 2G–I). *IRF3* expression, in contrast, demonstrated early stage sensitivity to ON-GNP treatment in HEL and SET2 cells at 2 h with *JAK2*V617F-GNP (~ 13-fold, p < 0.05) and at 1.5 h with *JAK2*-GNP (~ 30-fold, p < 0.0001), respectively. HEL cells exhibited a delayed but significant response at 48 h with *JAK2*V617F-GNP (*p* < 0.05) (Fig. 2J, K). In contrast, K562 cells exhibited early stage *IRF3* upregulation with *JAK2*-GNP treatment, starting at 0.5 h and persisting until 2 h, before diminishing at 24 h until 72 h. JAK2-GNP treatment induced IRF3 after (Fig. 2L).

The dynamics of *JAK2* expression following ON-GNP treatment underscored the complex regulatory mechanisms. Short-term suppression was followed by significant upregulation at 72 h in HEL cells treated with *JAK2*V617F-GNP (*p* < 0.01) indicating a potential compensatory mechanism in response to targeted suppression (Fig. 2M). In SET2 cells, *JAK2* expression significantly increased with *JAK2*-GNP at 24, 48, and 72 h (*p* < 0.0001, *p* < 0.001, and *p* < 0.0001, respectively) (Fig. 2N). Surprisingly, significant changes were observed in K562 cells (Fig. 2O), underscoring the specificity of ON-GNPs to *JAK2* contexts with early induction within 0.5–2 h following *JAK2*V617F-GNP and scramble-induced activation in 1.5–2 h. The *JAK2* activation diminished by 24 h and lasted until 72 h. Further studies are needed to elucidate whether ON-GNP treatment influences additional regulatory elements within the BCR-ABL and JAK2 signaling pathways.

This analysis demonstrates the intricate cellular responses to ON-GNPs and highlights the significant influence of genetic background and cell origin on gene expression dynamics. These findings suggest that ON-GNP-mediated interventions have the potential to modulate nucleic acid-sensing pathways and gene expression in a cell- and mutation-specific manner, providing insights into their utility as targeted therapeutic strategies in the context of MPNs.

### The effect of complementary JAK2V617F mRNAs attached gold nanoparticle stimulation on TLR9 and RAGE receptors

To investigate the mechanisms underlying the transient suppression and subsequent upregulation of TLR9 expression in response to ON-GNPs targeting the complementary transcripts of *JAK2*V617F, we analyzed the role of nucleic acid sensors, specifically TLR9 and RAGE. Nucleic acids and RAGE interact at the cell surface, promoting their uptake via the endosomal route, where the TLR9 receptors are predominantly located. The study aimed to distinguish the activation patterns of TLR9 and RAGE, hypothesizing that ON-GNPs may influence the TLR9/RAGE pathway and modulate the expression of other genes in a stimulus-duration-dependent manner.

**Fig. 3:**
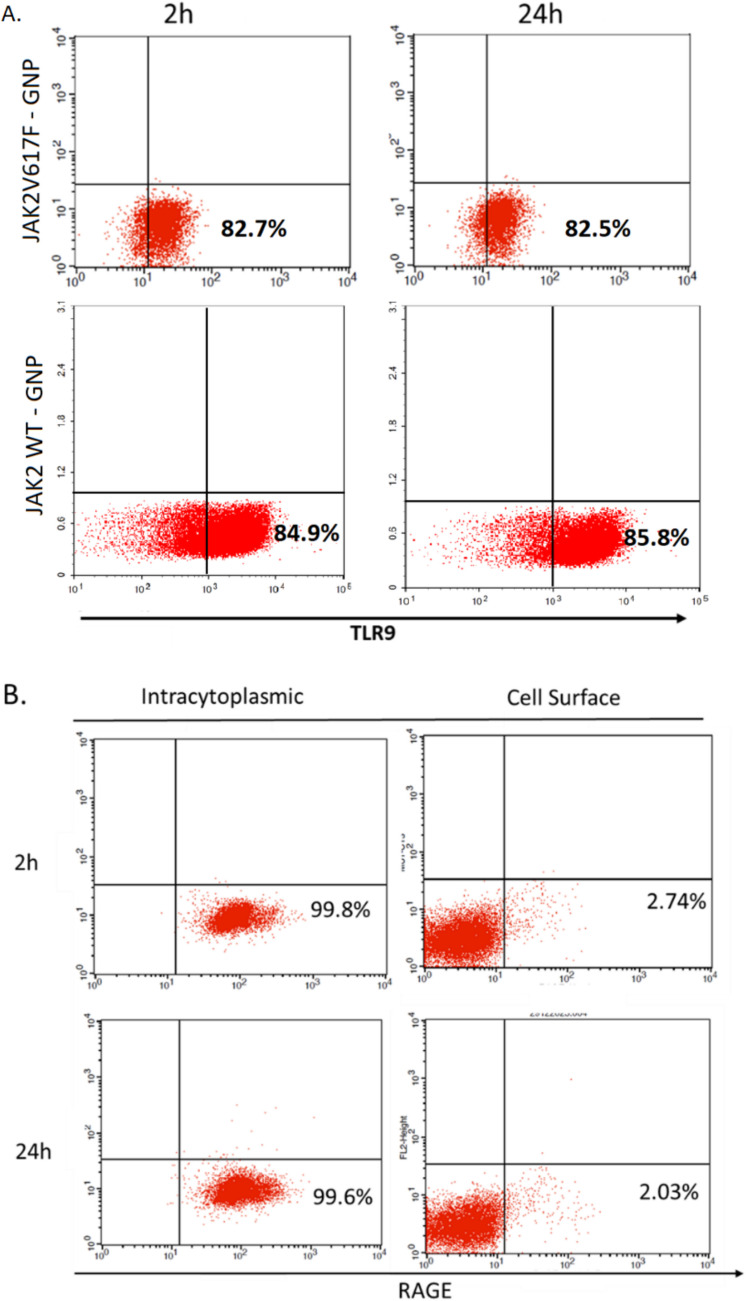
**A** Intracytoplasmic staining of TLR9 protein in HEL cells upon 2 h and 24 h incubation with *JAK2*V617F-GNPs and *JAK2*-GNPS by flow cytometer. **B** Intracytoplasmic and cell surface staining of RAGE protein for 2 h and 24 h incubation with JAK2V617F-GNP

Flow cytometry analysis of HEL cells incubated with *JAK2* and *JAK2*V617F-GNPs revealed no significant changes in intracytoplasmic TLR9 expression at either 2 h (short term) or 24 h (long term) of stimulation (Fig. 3A). Similarly, cell surface and intracytoplasmic RAGE protein levels remained unchanged after both short-term and long-term incubation with *JAK2*V617F-GNPs (Fig. 3B).

These findings highlight the complexity of TLR9 regulation and suggest that TLR9 expression observed in response to ON-GNPs may not directly involve RAGE-mediated pathway, indicating alternative mechanisms of regulation.

### The effect of complementary JAK2 and JAK2V617F mRNAs attached gold nanoparticle stimulation on inflammatory cytokines

**Fig. 4:**
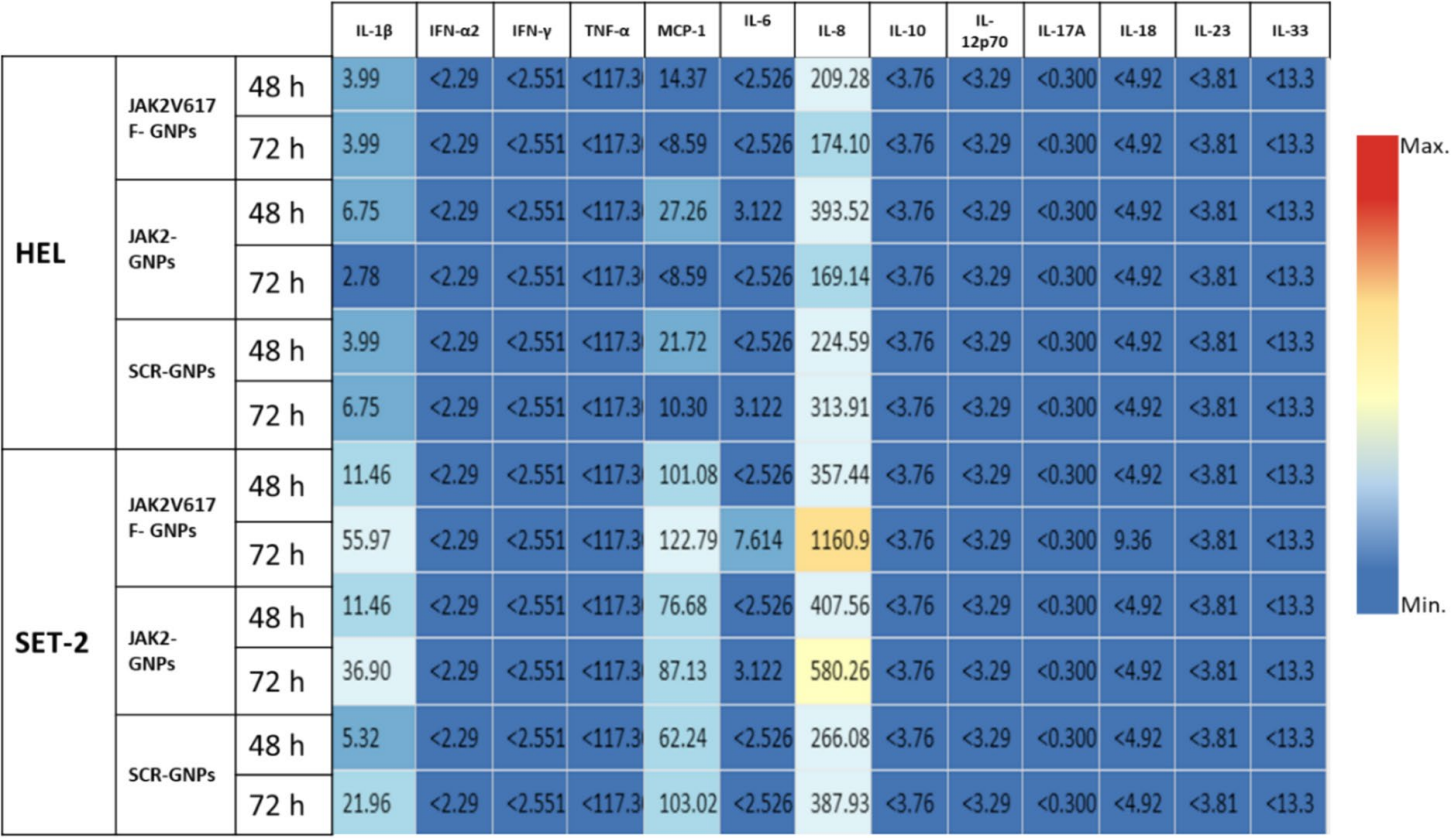
Inflammatory cytokine concentration levels of HEL and SET2 cells that were treated with JAK2 mRNA-specific, JAK2V617F mRNA-specific, and scrambled (non-specific to any mRNA)-GNPs detected with inflammation panel

To evaluate the impact of ON-GNPs on cytokine secretion in HEL and SET2 cells over extended periods, specifically at 24, 48, and 72 h, we employed a method that facilitated the quantification of various cytokines, including IL-1β, IFN-α2, IFN-γ, TNF-α, MCP-1, IL-6, IL-8, IL-10, IL-12p70, IL-17A, IL-18, IL-23, and IL-33, as shown in Fig. 4. Notably, a significant elevation in IL-8 levels was recorded in the HEL culture medium across all tested conditions, with the peak concentration observed at the 48-h mark following exposure to *JAK2*-GNPs. The cytokine profiles of SET2, presented in the figure, further complemented these findings.

## Discussion

This study introduces a novel therapeutic platform using ON-GNPs to modulate inflammatory pathways in Ph-MPNs. Our findings highlight the dynamic interplay between nucleic acid-sensing pathways, such as TLR9 and cGAS/STING, and inflammatory mediators like IRF7, while demonstrating the potential of ON-GNPs to recalibrate dysregulated immune responses.

Chronic inflammation is a hallmark of MPNs, contributing to genomic instability, clonal expansion, and fibrotic transformation (Karantanos and Moliterno 2018; Mendez Luque et al. 2019). The instability may also be exacerbated by extracellular vesicles, such as microparticles and exosomes, which can transfer genomic materials between cells, overloading recipient cytosols with DNA or RNA fragments (Li et al. 2021; Hekimoglu et al. 2022). The activation of TLR9 and cGAS-STING by cytosolic nucleic acids amplifies pro-inflammatory cytokine production, creating a feedback loop that perpetuates disease progression.

Our findings add to the growing evidence of the impact of *JAK2*V617F on nucleic acid-sensing pathways, particularly highlighting distinct patterns of TLR9 and IRF7 regulation. We observed initial suppression of *TLR9* expression following ON-GNP treatment, consistent with findings by Tsai et al. (Tsai et al. 2012), followed by significant upregulation at intermediate and late time points, especially in SET2 cells. This suggests that *JAK2*V617F-driven inflammation may be mediated, at least in part, through TLR9 and IRF7, both of which are central to innate immunity and inflammatory responses. Notably, our study is the first to establish a direct connection between *IRF7* and MPNs, positioning it as a critical mediator of type I interferon signaling in this context. *IRF7* was markedly upregulated in *JAK2*V617F-positive HEL and SET2 cells, underscoring its role in driving inflammation. The differential *IRF7* responses in HEL (erythroblastic origin) and SET2 (megaloblastic origin) cells reflect the distinct inflammatory profiles of polycythemia vera (PV) and essential thrombocythemia (ET). Elevated *IRF7* activity in HEL cells was associated with increased IL-8 secretion, a cytokine linked to thrombotic risk and leukocytosis in PV patients. In contrast, *IRF7* induction in SET2 cells was more subdued, consistent with the milder inflammatory phenotype observed in ET. The dual role of IRF7 in modulating both protective and pathogenic inflammation aligns with its established functions in other chronic inflammatory diseases and cancers (Qing and Liu 2023; Honda et al. 2005; Wang et al. 2022). Its activation downstream of TLR9 and cGAS/STING emphasizes the interconnected nature of these pathways, which are hyperactivated in MPNs. The ability of ON-GNPs to modulate *IRF7* provides a promising avenue for reducing cytokine-driven inflammation and mitigating disease progression.

The observed upregulation of *TLR9* gene expression following ON-GNP treatment, without a corresponding increase in protein levels, may be influenced by post-transcriptional and translational regulatory mechanisms or interactions with nucleic acid-binding proteins. Proteins such as HMGB1 or other RNA/DNA-binding factors could sequester ON-GNPs, interfering with their direct engagement with TLR9-related transcriptional or translational machinery (Tian et al. 2007; Ulloa and Messmer 2006; Gerstberger et al. 2014). These interactions might stabilize TLR9 mRNA while simultaneously inhibiting its efficient translation into protein, potentially due to competition for ON-GNP binding or altered mRNA transport and processing. This suggests a complex interplay between ON-GNPs, nucleic acid-binding proteins, and TLR9 regulation, requiring further investigation to fully elucidate these mechanisms.

The cGAS/STING pathway emerged as another key regulator of MPN-associated inflammation. Our findings show that prolonged ON-GNP exposure significantly increased *cGAS* expression across all three cell lines, while *STING* activation was selectively observed in HEL and SET2 cells. The delayed but robust cGAS upregulation in HEL and K562 cells, along with the selective activation of cGAS—but not *STING*—with non-specific GNP treatment in SET2 cells, suggests that *STING* activation does not necessarily follow *cGAS* induction, likely due to multiple regulatory mechanisms. One possible explanation is the post-translational regulation of STING, as STING undergoes ubiquitination, proteasomal degradation, and autophagic turnover, limiting its accumulation even in the presence of high cGAS expression (Xing et al. 2017). Another possibility is that *cGAS* may activate alternative inflammatory pathways, such as direct NF-κB signaling, bypassing STING-mediated responses (Neufeldt et al. 2022). This could explain why high *cGAS* expression coincides with increased *NFKB* activation, but not with *STING* induction. Additionally, epigenetic suppression of *STING* expression may contribute to this regulation. Recent studies suggest that MYC can directly bind to the STING enhancer region, repressing its transcription (Lee et al. 2022). Our previously published work (Uslu Bıçak et al. 2023) demonstrated elevated MYC expression in PV hematopoietic stem/progenitor cells which may contribute to *STING* suppression in these contexts. Moreover, we have also reported downregulation of the T-cell chemokines, including the CXCL9/CXCR3 axis, in PV (Altunay et al. 2018). Taken together, these findings highlight the intricate regulation of the cGAS/STING pathway in different hematopoietic malignancies. Our results suggest that STING activation is not a direct consequence of cGAS upregulation, but rather a highly cell-type-specific and context-dependent event. The accumulation of cytosolic DNA fragments, driven by genomic instability in *JAK2*V617F-positive cells, likely contributes to the activation of cGAS/STING and subsequent inflammatory responses. Interestingly, the selective activation of *IRF3* in SET2 cells suggests a context-dependent regulation of this pathway, influenced by lineage-specific factors (Yu et al. 2022; Liu et al. 2018; Wu et al. 2022). While cGAS/STING is known to exert anti-tumor effects by promoting immune responses, its dysregulation in MPNs may drive excessive inflammation and fibrosis. The ability of ON-GNPs to indirectly modulate cGAS/STING highlights their potential as therapeutic tools for addressing inflammation and genomic instability in MPNs.

Cytokine analysis revealed significant IL-8 upregulation in HEL cells following ON-GNP treatment, aligning with its established role in MPN-related inflammation and disease burden (Kleppe et al. 2018; Dunbar et al. 2023; Pallares et al. 2019; József et al. 2006; Di et al. 2009). Elevated IL-8 levels are associated with leukocytosis, thrombosis, and fibrosis, particularly in PV (Dunbar et al. 2023; Masselli et al. 2020). The ability of ON-GNPs to modulate IL-8 and other cytokines underscores their relevance in addressing both the inflammatory and proliferative components of MPNs. The lack of consistent correlation between cGAS/STING activation and cytokine release, particularly IL-8 and TNF-α, suggests the involvement of alternative pathways or cytoplasmic shuttling molecules interacting with ON-GNPs (Gerstberger et al. 2014; Aitken et al. 2022). RAGE, a receptor implicated in nucleic acid uptake and TLR9 activation, showed no significant changes in our study, suggesting that alternative mechanisms may regulate TLR9-mediated inflammation in MPNs. This aligns with recent findings that RAGE-independent pathways can drive chronic inflammation through TLR9 and related sensors (Rojas et al. 2024).

**Fig. 5:**
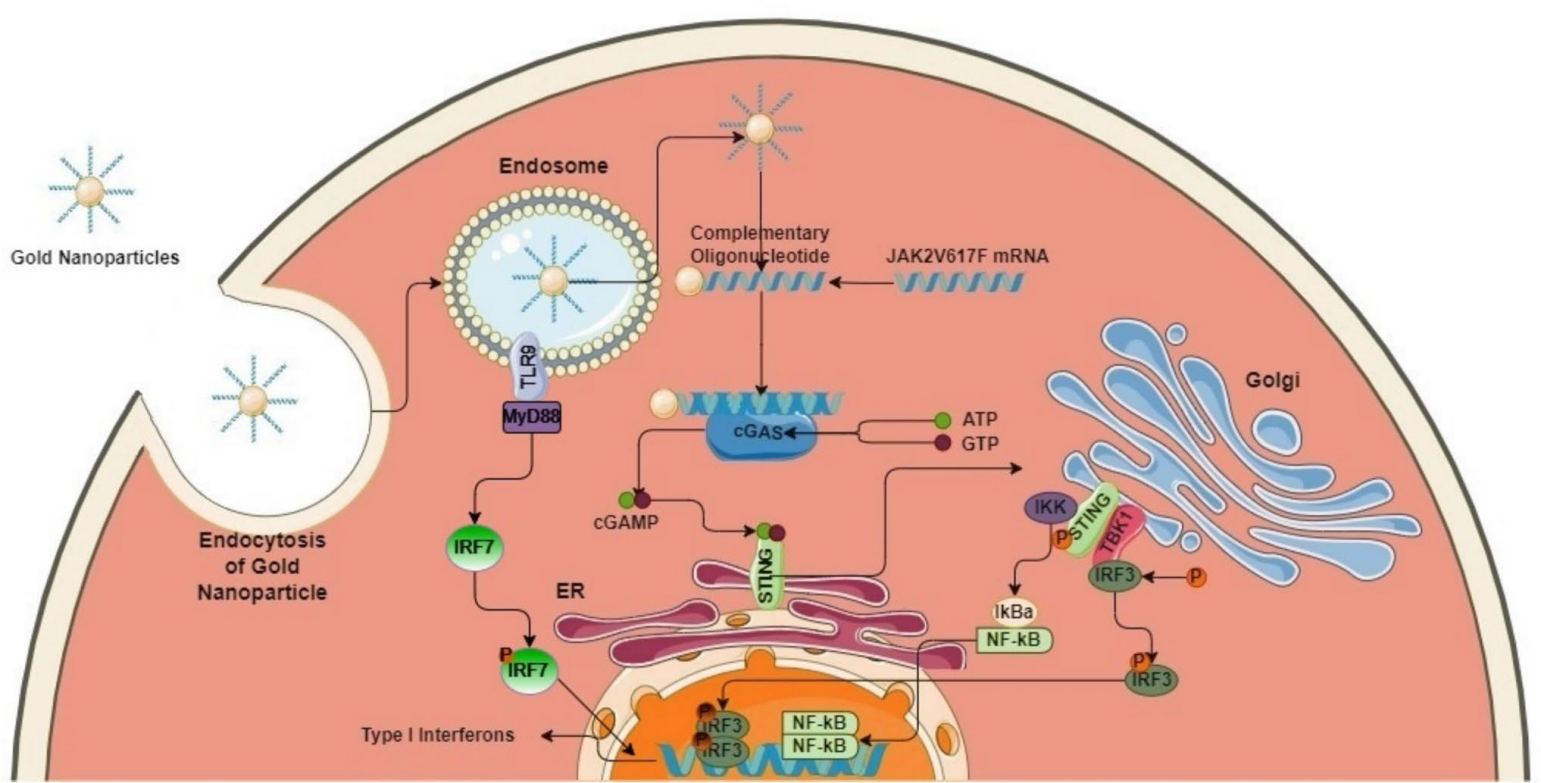
Illustration of the mechanism of ON-GNPs and their effects on the cGAS/STING and TLR9 pathways

ON-GNPs represent a precision therapy capable of targeting nucleic acid-sensing pathways while modulating cytokine production. Their specificity and adaptability make them particularly suited for addressing the unique inflammatory profiles of MPN subtypes. In PV, ON-GNPs targeting TLR9, IRF7, and IL-8 pathways may reduce thrombotic risk and leukocytosis, while in ET, they could mitigate platelet activation and chronic inflammation. The role of ON-GNPs in recalibrating nucleic acid-sensing pathways also positions them as potential adjuncts to JAK inhibitors, enhancing their anti-inflammatory effects. Furthermore, their ability to engage both TLR9 and cGAS/STING pathways highlights their multifaceted therapeutic potential in inflammation-driven malignancies as illustrated in Fig. 5.

## Conclusion

This study establishes a novel link between IRF7 and MPN pathophysiology while demonstrating the potential of ON-GNPs to modulate key inflammatory pathways by targeting nucleic acid-sensing mechanisms, including TLR9 and cGAS/STING; ON-GNPs recalibrate dysregulated inflammation and mitigate cytokine-driven pathology in MPNs. These findings provide a foundation for future investigations into ON-GNP-based therapies, offering a precision medicine approach for managing MPNs and related inflammatory conditions.

## Supplementary Information

Below is the link to the electronic supplementary material.

Supplementary file1 (DOCX 13 KB)

## Data Availability

The data is available upon request.
