# Supplementary material for: Suppressed activation of the IRF7 and TLR9 by JAK2V617F gold nanoparticles
Source: Immunogenetics. 2025 Feb 28;77(1):16. doi: 10.1007/s00251-025-01374-y (PMC11868351; doi:10.1007/s00251-025-01374-y)
Supplement: Supplementary file 1 — Supplementary file1 (DOCX 13 KB) [file 251_2025_1374_MOESM1_ESM.docx]

*Supplement Table* 1: Primer sequences

| **Gene** | **Primer Sequences** | |
| --- | --- | --- |
| ON-GNP | *JAK2* | 5’ TCT CCA CAG ACA CAT ACT CCA 3’ |
|  | *JAK2*V617F | 5’ TCT CCA CAG AAA CAT ACT CCA 3’ |
|  | *Scramble* | 5’ TGG TAC ATT CGG CTA TCC AGC GCC 3’ |
| *cGAS* | Forward | 5’ TAA CCC TGG CTT TGG AAT CAA AA 3’ |
|  | Reverse | 5’TGG GTA CAA GGT AAA ATG GCT TT 3’ |
| *STING* | Forward | 5’GAG CAG GCC AAA CTC TTC TG 3’ |
|  | Reverse | 5’TGC CCA CAG TAA CCT CTT CC 3’ |
| *JAK2* | Forward | 5’CCA GAT GGA AAC TGT TCG CTC AG 3’ |
|  | Reverse | 5’GAGGTTGGTACATCAGAAACACC 3’ |
| *TBK1* | Forward | 5’ CAA CCT GGA AGC GGC AGA GTT A 3’ |
|  | Reverse | 5’ ACC TGG AGA TAA TCT GCT GTC GA 3’ |
| *IRF3* | Forward | 5’ TCT GCC CTC AAC CGC AAA GAA G 3’ |
|  | Reverse | 5’ TAC TGC CTC CAC CAT TGG TGT C 3’ |
| *TLR9* | Forward | 5’ TGA GCC ACA ACT GCA TCT CGC A 3’ |
|  | Reverse | 5’-CAG TCG TGG CTC CGT GAA T 3’ |
| *IRF7* | Forward | 5’ CCA CGC TAT ACC ATC TAC CTG G 3’ |
|  | Reverse | 5’ GCT GCT ATC CAG GGA AGA CAC A 3’ |
| *NFKB1* | Forward | 5’ GCA GCA CTA CTT CTT GAC CAC C 3’ |
|  | Reverse | 5’ TCT GCT GAG CAT TGA CGT C-3’ |
| *ACTB* | Forward | 5’ CCA ACC GCG AGA AGA TGA 3’ |
|  | Reverse | 5’ CCA GAG GCG TAC AGG GAT AG 3’ |
